# Racial and ethnic differences and the role of unfavorable social determinants of health across steatotic liver disease subtypes in the United States

**DOI:** 10.1097/HC9.0000000000000324

**Published:** 2023-12-01

**Authors:** Pedro Ochoa-Allemant, Jorge A. Marrero, Marina Serper

**Affiliations:** 1 Division of Gastroenterology and Hepatology, Department of Medicine, Perelman School of Medicine, University of Pennsylvania, Philadelphia, Pennsylvania, USA; 2 Leonard Davis Institute of Health Economics, University of Pennsylvania, Philadelphia, Pennsylvania, USA; 3 Corporal Michael J. Crescenz VA Medical Center, US Department of Veterans Affairs, Philadelphia, Pennsylvania, USA

## Abstract

**Background:**

The global liver community established a more precise criteria to characterize steatotic liver disease (SLD), specifically metabolic dysfunction–associated steatotic liver disease (MASLD) and metabolic dysfunction–associated and alcohol-associated liver disease (MetALD). We aimed to estimate the burden of SLD subtypes and unfavorable social determinants of health (SDOH) in US adults and whether clinical and social factors drive disparities across racial/ethnic subgroups.

**Methods:**

We evaluated 4263 persons aged 20 years or older from the National Health and Nutrition Examination Survey 2017–2018. We estimated the weighted age-adjusted prevalence and severity of SLD, examined the prevalence of SDOH across SLD subtypes, and performed stepwise regression analysis to evaluate associations between race/ethnicity and SLD, accounting for metabolic risks, alcohol use, and SDOH.

**Results:**

Hispanic adults had the highest prevalence of MASLD (22.3%), MASLD-predominant MetALD (10.3%), alcohol-associated liver disease (ALD)-predominant MetALD (5.6%), and ALD (5.4%). Hispanic adults with MASLD had the highest prevalence of high-risk metabolic dysfunction–associated steatohepatitis (18.0%) and advanced fibrosis (21.1%), whereas non-Hispanic (NH) White adults with MetALD had the highest prevalence of high-risk metabolic dysfunction–associated steatohepatitis (19.3%), advanced fibrosis (19.5%), and cirrhosis (8.1%). Adults with ALD-predominant MetALD and ALD had an increased burden of unfavorable SDOH than those with MASLD, particularly food insecurity, limited health care access, and single living. In stepwise regression, the odds of SLD in Hispanic adults decreased after adjusting for metabolic risks (OR 1.40, 95% CI, 1.06–1.84) and alcohol use (OR 1.36, 95% CI, 1.01–1.82). Differences did not persist after adjusting for cumulative SDOH and nativity status (OR 1.22, 95% CI, 0.89–1.68).

**Conclusions:**

We found substantial disparities in the burden of unfavorable SDOH across SLD subtypes, particularly among those with ALD-predominant MetALD and ALD. Population-based approaches targeting SDOH may mitigate racial/ethnic differences among US adults with SLD.

## Graphical abstract

**Figure:**
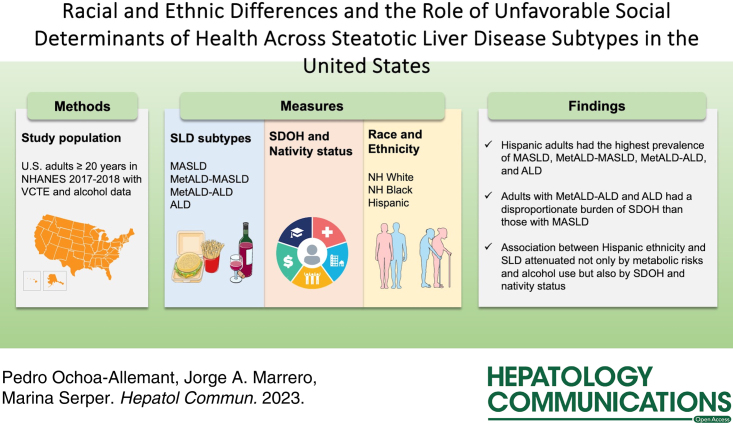

## INTRODUCTION

The global burden of hepatic steatosis, formerly known as NAFLD, is substantial and increasing at an alarming rate, with a projected global prevalence of 55% in 2040.^1^ In the United States, at least 1 in 4 adults have hepatic steatosis, with an annual direct medical cost of about 103 billion dollars.^2,3^ Given this growing public health concern and the consensus need for an affirmative, nonstigmatizing name and diagnosis,^4^ the global liver community, including clinicians, researchers, industry, and patient advocacy groups, convened to establish a more precise definition for NAFLD in June 2023.^5^

The new multi-society nomenclature creates a subclassification system, replaces the term “fatty” with steatotic, and incorporates cardiometabolic risk factors (CMRFs) and alcohol consumption into its definitions.^5^ Specifically, metabolic dysfunction–associated steatotic liver disease (MASLD) and metabolic dysfunction–associated and alcohol-associated liver disease (MetALD) to describe those with MASLD and heavy alcohol use. Despite the introduction of these subcategories to better characterize steatotic liver disease (SLD), few data exist on the burden and severity of SLD subtypes according to the new nomenclature in the US population. For instance, a recent study using the National Health and Nutrition Examination Survey (NHANES) aimed to define SLD^6^ however, thorough characterization of SLD subtypes such as MASLD-predominant and alcohol-associated liver disease (ALD)-predominant metabolic dysfunction–associated and alcohol-associated liver disease (MetALD), evaluation of the severity of SLD, or investigation of the impact of risk factors such as social determinants of health (SDOH) is lacking.

Health disparities in what was previously termed NAFLD have been well documented in the United States, especially in racialized groups.^7,8^ Although race and ethnicity are both a social and political construct, grouping people based on skin color and, to some extent, shared heritage, has shaped people’s experiences and access to resources that have determined disparities in morbidity and mortality.^9,10^ For instance, individuals who identified their origin as Hispanic have a substantial higher prevalence of hepatic steatosis and higher odds of progression to end-stage liver disease and HCC,^7^ yet few studies have characterized disparities in prognosis.^11^ Furthermore, data shows that racialized groups are disproportionately affected by uncertain access to adequate food, are targeted by unhealthy food and alcohol advertising, and have limited access to health care, leading to a decrease use of preventive services.^8^ Nonetheless, the impact of underlying social risk factors affecting these populations with SLD is scarce.^12–14^

To fill current knowledge gaps, the aims of our study were to (1) define SLD subtypes, namely MASLD, MetALD, and ALD, in the US population according to new nomenclature; (2) estimate and compare population-based, age-adjusted prevalence and severity of SLD subtypes among US adults and across racial and ethnic subgroups; (3) examine the prevalence of unfavorable SDOH by SLD subtypes and race/ethnicity; and (4) evaluate how medical comorbidities, alcohol use, and SDOH moderate associations between race/ethnicity and SLD.

## METHODS

### Data source and patient population

In this cross-sectional study, we used data from the NHANES 2017–2018. NHANES is a population-based survey that incorporates a nationally representative sample of the US population conducted by the Centers for Disease Control and Prevention National Center for Health Statistics.^15^ Information collected includes interviews, physical examinations, and laboratory tests, and the cycle 2017–2018 obtained vibration-controlled transient elastography (VCTE) information.^16^ The National Center for Health Statistics Institutional Review Board approved the study protocols, and all the participants provided written informed consent. Our study population included adults aged 20 years or older who completed a VCTE exam. We restricted our data to those with a reliable VCTE exam and complete alcohol use information.

### Study variables and definitions

We obtained self-reported information on age, gender, and race/ethnicity. Racial and ethnic subgroups included non-Hispanic (NH) White, NH Black, Hispanic (including Mexican American and other Hispanic), and NH other (including NH Asian and multiracial) adults. Other Hispanic adults included all individuals self-identifying as Hispanic but not Mexican American.

We evaluated SDOH measures aligned with the Healthy People 2030 SDOH five domains, namely (1) economic stability; (2) education access and quality; (3) health care access and quality; (4) neighborhood and built environment; and (5) social and community context.^17–20^ Employment status (economic stability) was divided into unemployed and employed, student, or retired. Household income (economic stability) was calculated as the ratio of family income to poverty level and dichotomized at 130%, the limit to be eligible for several federal programs such as the Supplemental Nutrition Assistance Program. Food security (economic stability) was determined based on responses to 10 questions of the US Food Security Survey Module and categorized as full security (no affirmative response) or food insecurity (marginal, low, or very low security).^21^ Education level (education access and quality) was divided into high school graduate or less and some college or higher. Health insurance (health care access and quality) was grouped as private and public/none. Access to health care (health care access and quality) was based on whether a participant had a routine facility to access health care other than an emergency room. Home ownership (neighborhood and built environment) was divided as owned and rented or other arrangements. Marital status (social and community context) was grouped as married or living with a partner and not married nor living with a partner (widowed, divorced, separated, or never married). A cumulative SDOH score was created by adding the 8 dichotomized SDOH variables, assigning a value of 0 for each favorable and 1 for each unfavorable level.^19,20^ Given the small proportion of participants reporting 6, 7, or 8 unfavorable SDOH variables, they were combined, and the cumulative SDOH score ranged from 0 (all favorable) to 6 or more (unfavorable). We also obtained nativity status categorized as US-born and foreign-born.

Comorbidities of interest included current cigarette smoking, diabetes (fasting plasma glucose ≥ 126 mg/dL, hemoglobin A1c ≥ 6.5%, or use of insulin or oral antidiabetic medications), hypertension (mean blood pressure ≥130/80 mm Hg or use of antihypertensive medications), dyslipidemia (HDL cholesterol ≤ 40 mg/dL for men and ≤ 50 mg/dL for women, LDL cholesterol ≥130, triglycerides ≥ 200 mg/dL, total cholesterol ≥ 200 mg/dL or the use of lipid-lowering treatment), BMI, and waist circumference. We extracted lifetime and current alcohol use information (past 12 mo) and defined alcohol consumption as alcohol drinks per day (eg, 0 drinks per day was assigned if participants denied drinking alcohol in their entire lifetime or in the past 12 mo).

We identified the variables included in the proposed 5 adult CMRF as follows: (1) central obesity (waist circumference >102 cm for men and >88 cm for women); (2) insulin resistance (fasting serum glucose ≥100 mg/dL, hemoglobin A1c ≥5.7%, type 2 diabetes, or use of antidiabetic treatment); (3) elevated blood pressure (≥130/85 mm Hg or antihypertensive treatment); (4) hypertriglyceridemia (triglycerides ≥150 mg/dL or the use of lipid-lowering treatment); and (5) reduced HDL cholesterol (≤ 40 mg/dL for men and ≤ 50 mg/dL for women or the use of lipid-lowering treatment).^5^ Of note, we followed the waist circumference thresholds recommended for the US population according to the guidelines of the National Cholesterol Education Program Adult Treatment Panel III and the American Heart Association.^22,23^

We calculated the FibroScan-aspartate aminotransferase score using liver stiffness measurement (LSM), controlled attenuation parameter, and aspartate aminotransferase.^24^ We also obtained the fibrosis-4 score and NAFLD fibrosis score as described,^25,26^ considering the optimized cutoffs for patients aged ≥ 65 years.^27^

### Definition of SLD subtypes and disease severity measures

We used a controlled attenuation parameter ≥ 285 dB/m (cutoff optimizing sensitivity and specificity) to identify hepatic steatosis.^28^ We defined MASLD and MetALD with predominant MASLD or ALD as outlined in the multi-society consensus statement.^5^ Specifically, MASLD was defined as (1) hepatic steatosis; (2) ≥ 1 CMRF; and (3) < 2 drinks/day for women and < 3 drinks/day for men. MetALD was defined as (1) hepatic steatosis; (2) ≥ 1 CMRF; and (3) ≥ 2 drinks/day for women and ≥ 3 drinks/day for men. MASLD-predominant MetALD was defined as MetALD with 2–3 drinks/day for women and 3–4 drinks/day for men. ALD-predominant MetALD was defined as MetALD with > 3 drinks/day for women and > 4 drinks/day for men. We used the threshold of > 3 drinks/day for women and > 4 drinks/day for men, given the definition of heavy alcohol use by the National Institute on Alcohol Abuse and Alcoholism.^29^ We identified ALD based on heavy alcohol use (>3 drinks/day for women and >4 drinks/day for men) associated with (1) hepatic steatosis in the absence of CMRFs or (2) elevated aminotransferases (aspartate aminotransferase or alanine transaminase > 25 IU/L in women and >35 IU/L in men) in the absence of elevated total bilirubin (<3 mg/dL) and after excluding hepatitis C and hepatitis B infections. The latter was included as this is the definition of the latest US clinical guideline that has been used in epidemiological studies.^30,31^

We used a FibroScan-aspartate aminotransferase score ≥ 0.35 to identify high-risk metabolic dysfunction–associated steatohepatitis (MASH).^24,32^ We defined advanced fibrosis and cirrhosis as an LSM ≥ 8.6 kPa and ≥13.1 kPa (cutoffs optimizing sensitivity and specificity), respectively.^28^

### Statistical analysis

First, we obtained population-based estimates and calculated the age-adjusted prevalence of hepatic steatosis, MASLD, MetALD, and ALD for all US adults and across racial and ethnic subgroups. We performed age adjustment applying the direct method to the 2000 US Census population as the reference using the age groups 20–39, 40–59, and 60 years and over.^33^ We then examined the age-adjusted prevalence of high-risk MASH, advanced fibrosis, and cirrhosis among those with MASLD and MetALD by race and ethnicity. Next, we evaluated the prevalence of unfavorable SDOH across SLD subtypes and race/ethnicity. Lastly, we developed a stepwise logistic regression analysis to determine the associations between the primary exposures of race and ethnicity and SLD. Model 1 included demographic characteristics (age, sex). Model 2 included variables of model 1 and metabolic risks (hypertension, diabetes, BMI). Model 3 included variables from model 2 and alcohol consumption (drinks/day). Model 4 included variables from model 3 as well as the cumulative SDOH score and nativity status.

We conducted all analyses using Stata statistical software, version 18.0 (StataCorp, College Station, TX). We used survey analysis procedures to account for the complex sampling design and obtain nationally representative estimates. All 95% CI and *p* values were based on two-sided hypothesis tests, for which *p* < 0.05 was considered statistically significant.

## RESULTS

### Study population

**FIGURE 1:**
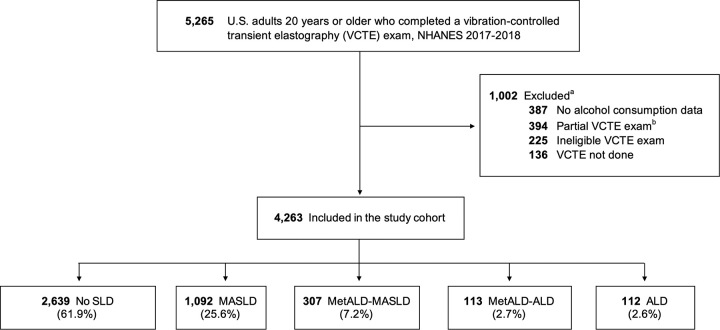
Study population flow chart. Unweighted total number of participants.^a^ Some patients presented with more than one excluding criteria.^b^ Partial exam includes fasting < 3 h, unable to obtain 10 valid measurements, and IQR/M > 30%. Abbreviations: ALD, alcohol-associated liver disease; MASLD, metabolic dysfunction–associated steatotic liver disease; MetALD, metabolic dysfunction–associated and alcohol-associated steatotic liver disease; NHANES, National Health and Nutrition Examination Survey; VCTE, vibration-controlled transient elastography.

We identified a total of 4263 persons aged 20 years or older who underwent a reliable VCTE and had complete alcohol use information in the NHANES 2017–2018 (Figure 1), representing a total of 199.1 million adults in the United States. Overall, participants had a weighted mean (SD) age of 48 (15) years, and 50.3% were women. This group included 63.0% NH White adults, 11.1% NH Black adults, 15.8% Hispanic adults (8.9% Mexican American, 6.9% other Hispanic), and 10.2% comprised other race/ethnicity (including NH Asian and multiracial adults). Among patients with SLD, adults with pure MASLD were more likely to have metabolic risk factors such as hypertension (72.9%) and diabetes (32.0%), whereas adults with ALD were more likely to be younger (20–39 years, 54.0%) and from Hispanic origin (30.9%). Further comparisons of weighted clinical characteristics are presented for all US adults and across racial and ethnic subgroups in Table 1 and Supplemental Table S1, http://links.lww.com/HC9/A666, respectively.

**TABLE 1:** Weighted clinical characteristics of US adults 20 years or older by SLD subtypes, NHANES 2017–2018

	Weighted, % (95% CI)				
			MetALD^a^		
	Overall population	MASLD^b^	MASLD-Pred^c^	ALD-Pred^d^	ALD^e^
Demographics					
Population estimate	199,149,561	44,888,618	16,897,094	5,062,075	5,892,916
Age, mean (SD), y	48 ± 15	55 ± 14	47 ± 12	42 ± 13	39 ± 10
Age, group, y					
20–39	36.2 (32.8–39.7)	18.0 (15.6–20.8)	33.8 (26.8–41.5)	46.5 (32.3–61.3)	54.0 (38.2–69.0)
40–59	35.3 (32.3–38.4)	39.6 (32.6–47.0)	41.1 (33.0–49.7)	34.3 (20.3–47.2)	42.2 (29.6–55.8)
≥60	28.5 (24.9–32.4)	42.4 (36.9–48.0)	25.2 (17.7–34.5)	19.2 (9.8–34.4)	3.90 (1.2–12.0)
Gender, women	50.3 (48.3–52.3)	40.9 (35.6–46.3)	56.3 (49.0–63.3)	31.9 (22.1–43.6)	37.2 (24.6–51.7)
Race and ethnicity					
NH White	63.0 (57.5–68.3)	66.3 (59.9–72.1)	63.1 (52.6–72.5)	56.8 (39.1–72.9)	50.4 (36.6–64.1)
NH Black	11.1 (8.1–15.0)	8.9 (6.1–12.7)	8.8 (5.2–14.5)	4.5 (1.7–11.8)	8.1 (3.2–19.2)
All Hispanic	15.8 (12.2–20.1)	14.2 (11.0–18.2)	19.3 (12.1–29.3)	27.5 (22.1–55.9)	30.9 (21.8–41.7)
Mexican American	8.9 (6.1–12.8)	8.5 (5.9–12.2)	13.8 (76–23.6)	31.3 (17.5–49.6)	21.7 (14.4–31.3)
Other Hispanic	6.9 (5.3–8.8)	5.7 (4.2–7.7)	5.5 (2.5–11.5)	6.1 (3.4–10.7)	9.2 (4.6–17.7)
NH Other	10.2 (7.8–13.1)	10.6 (7.3–15.2)	8.9 (5.3–14.6)	1.3 (0.3–4.6)	10.6 (5.2–20.6)
Risk factors					
Cigarette smoking	17.2 (14.6–20.1)	9.2 (7.3–11.5)	24.4 (18.9–30.8)	36.8 (22.6–53.7)	32.3 (20.5–46.9)
Alcohol consumption, mean (SD), drinks/day	1.9±1.9	0.8±0.7	2.8±0.6	6.4±2.3	6.7±2.5
Dyslipidemia	66.7 (63.0–70.2)	81.3 (74.1–86.9)	83.8 (77.2–88.7)	75.5 (61.6–85.5)	59.1 (48.1–69.3)
Hypertension	51.0 (47.7–54.3)	72.9 (68.0–77.2)	65.0 (54.5–74.2)	54.6 (42.1–66.6)	57.6 (44.9–69.4)
Diabetes	13.4 (12.3–14.7)	32.0 (28.9–35.3)	18.9 (12.7–27.2)	10.9 (6.8–17.1)	15.4 (7.0–30.6)
BMI, mean (SD), kg/m^2^	29.6±6.1	33.9±6.2	34.5±5.7	35.6±5.0	32.5±6.6
Waist circumference, mean (SD), cm	100±15	112±13	112±12	115±12	107±15
HOMA-IR	3.8±6.1	6.7±11.0	4.9±4.3	4.3±3.1	6.1±6.8
AST, mean (SD), IU/L	22±11	22±9	25±14	18±4	41±23
ALT, mean (SD), IU/L	23±15	26±14	32±22	21±7	48±20
Total energy, mean (SD), kcal/day	2,105±750	2,126±777	2,126±594	1,975±690	2,531±783
Total sugar, mean (SD), gm	104±58	107±68	100±43	85±43	114±58
CAP, mean (SD), dB/m	264±54	331±30	330±28	333±30	304±52
LSM, mean (SD), kPa	5.7±4.1	6.9±4.8	6.7±5.3	7.2±6.9	7.6±8.8
FAST score, mean (SD)	0.12±0.13	0.18±0.16	0.20±0.16	0.12±0.13	0.34±0.19
FIB-4 score >2.67	2.3 (1.7–3.2)	3.0 (2.1–4.4)	0.5 (0.2–1.9)	N/A^f^	6.1 (2.1–16.7)
NFS score >0.676	8.1 (6.8–9.7)	16.5 (13.7–19.8)	7.9 (4.4–13.8)	4.8 (2.0–11.0)	9.8 (2.8–29.4)

a MetALD was defined as (1) hepatic steatosis; (2) ≥ 1 CMRF; and (3) ≥ 2 drinks/day for women and ≥ 3 drinks/day for men.

b MASLD was defined as (1) hepatic steatosis; (2) ≥ 1 CMRF; and (3) < 2 drinks/day for women and < 3 drinks/day for men.

c MASLD-predominant MetALD was defined as MetALD with 2–3 drinks/day for women and 3–4 drinks/day for men.

d ALD-predominant MetALD was defined as MetALD with > 3 drinks/day for women and > 4 drinks/day for men.

e ALD was defined as > 3 drinks/day for women and > 4 drinks/day with hepatic steatosis in the absence of CMRF and/or AST or ALT > 35 IU/L for men and > 25 IU/L for women in the absence of HBV or HCV infection.

f Unable to be analyzed because of the insufficient number of participants.

Abbreviations: ALD, alcohol-associated liver disease; CAP, controlled attenuation parameter; CMRF, cardiometabolic risk factor; FAST, FibroScan-aspartate aminotransferase; FIB-4, fibrosis-4; HOMA-IR, Homeostatic Model Assessment for Insulin Resistance; LSM, liver stiffness measurement; MASLD, metabolic dysfunction–associated steatotic liver; MetALD, metabolic dysfunction–associated and alcohol-associated liver disease; NFS, nonalcoholic fatty liver disease fibrosis score; NH, non-Hispanic; NHANES, National Health and Nutrition Examination Survey.

### Prevalence and severity of SLD subtypes

**FIGURE 2:**
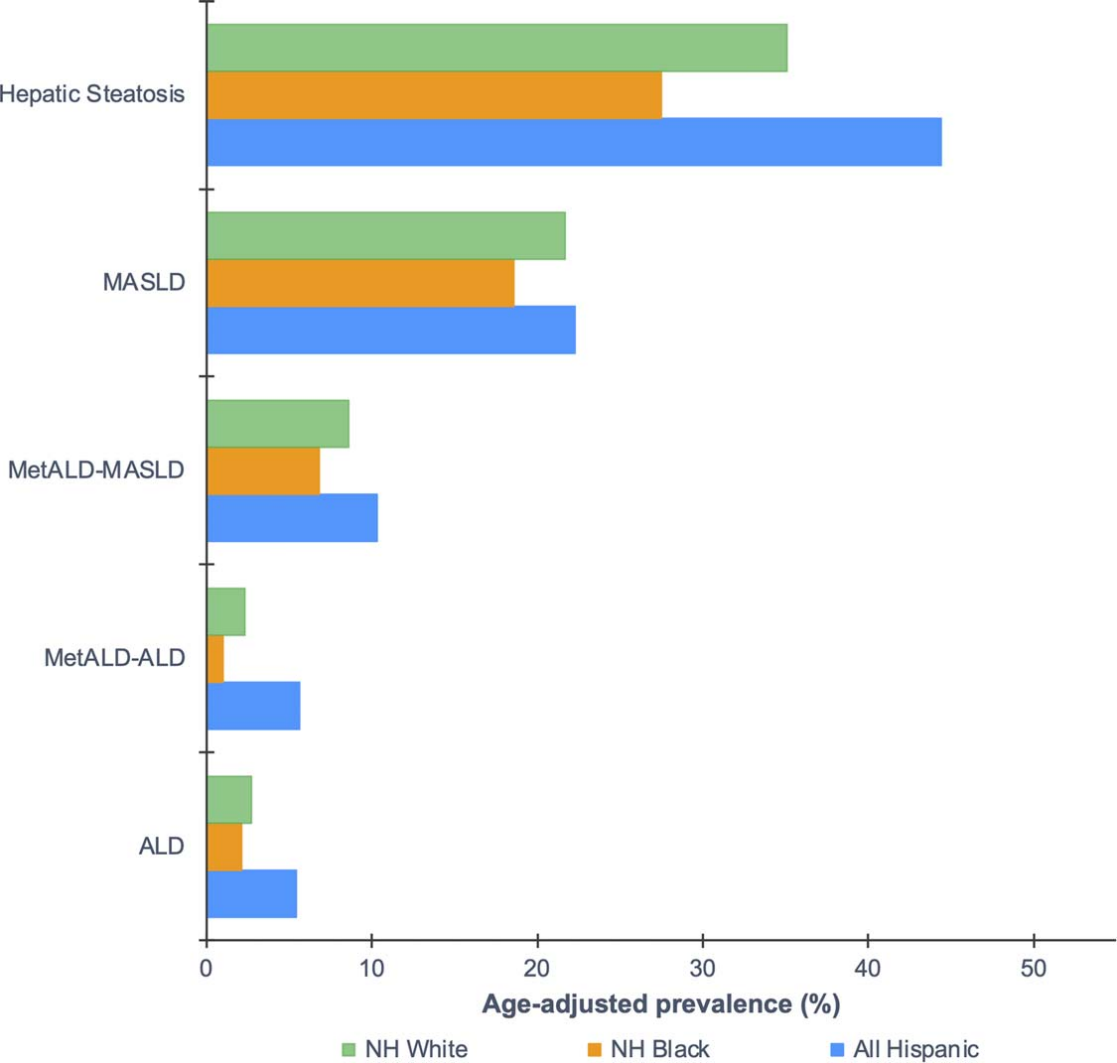
Weighted age-adjusted prevalence of hepatic steatosis and SLD among US adults 20 years or older by race and ethnicity, NHANES 2017–2018. Estimates were adjusted by the direct method to the 2000 US Census population using age groups 20–30, 40–59, and 60 and over. Hepatic steatosis was defined as a CAP ≥ 285 dB/m. MASLD was defined as (1) hepatic steatosis; (2) ≥ 1 CMRF; and (3) < 2 drinks/day for women and < 3 drinks/day for men. MetALD was defined as (1) hepatic steatosis; (2) ≥ 1 CMRF; and (3) ≥ 2 drinks/day for women and ≥ 3 drinks/day for men. MASLD-predominant MetALD was defined as MetALD with 2–3 drinks/day for women and 3–4 drinks/day for men. ALD-predominant MetALD was defined as MetALD with > 3 drinks/day for women and > 4 drinks/day for men. ALD was defined as > 3 drinks/day for women and > 4 drinks/day with hepatic steatosis in the absence of CMRF and/or AST or ALT > 35 IU/L for men and >25 IU/L for women in the absence of HBV or HCV infection. Abbreviations: ALD, alcohol-associated liver disease; CAP, controlled attenuation parameter; CMRF, cardiometabolic risk factors; MASLD, metabolic dysfunction–associated steatotic liver; MetALD; metabolic dysfunction–associated and alcohol-associated liver disease; NH, non-Hispanic; NHANES, National Health and Nutrition Examination Survey; SLD, steatotic liver disease.

Of the estimated US population, 72.4 million adults had hepatic steatosis, 44.9 million adults had pure MASLD, 16.9 million adults had MASLD-predominant MetALD, 5.1 million adults had ALD-predominant MetALD, and 5.9 million adults had ALD. The age-adjusted prevalence of hepatic steatosis was 35.7%, MASLD was 22.6%, MetALD was 11.2% (MASLD-predominant 8.5%, ALD-predominant 2.6%), and ALD was 3.2% (Figure 2). Across racial and ethnic groups, Hispanic adults had the highest age-adjusted prevalence of hepatic steatosis (44.4%), MASLD (22.3%), MASLD-predominant MetALD (12.6%), ALD-predominant MetALD (8.0%), and ALD (6.5%). These differences were substantially higher in Mexican-American adults (Supplemental Table S2, http://links.lww.com/HC9/A666).

**FIGURE 3:**
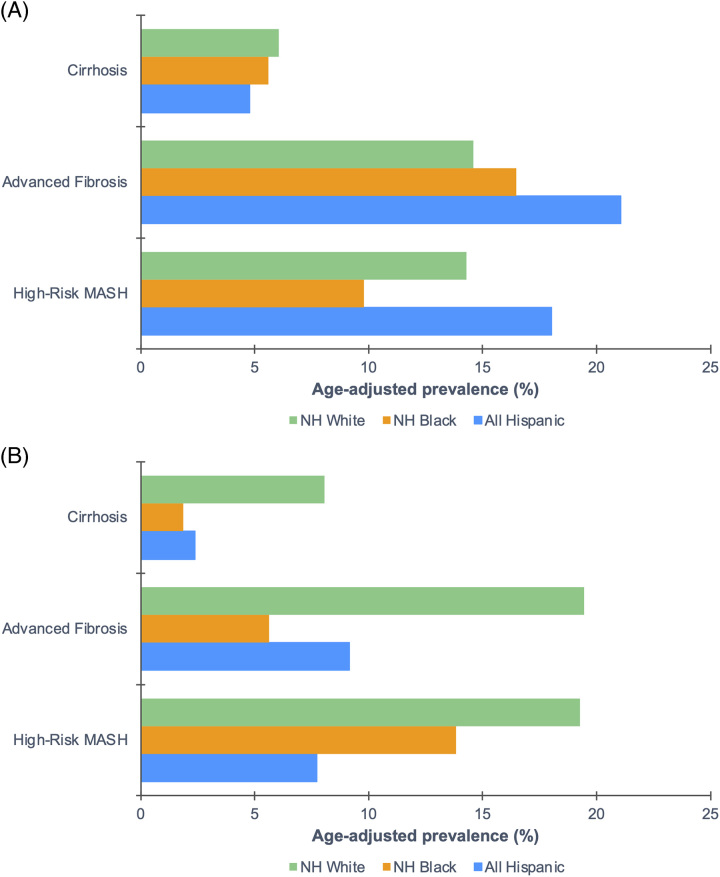
Weighted age-adjusted prevalence of high-risk MASH, advanced fibrosis, and cirrhosis among US adults 20 years or older with MASLD and MetALD by race and ethnicity, NHANES 2017–2018. (A) Individuals with MASLD. (B) Individuals with MetALD. Estimates were adjusted by the direct method to the 2000 US Census population using age groups 20–30, 40–59, and 60 and over. MASLD was defined as (1) hepatic steatosis; (2) ≥ 1 CMRF; and (3) < 2 drinks/day for women and < 3 drinks/day for men. MetALD was defined as (1) hepatic steatosis; (2) ≥ 1 CMRF; and (3) ≥ 2 drinks/day for women and ≥ 3 drinks/day for men. High-risk MASH was defined as FAST score ≥ 0.35. Advanced fibrosis was defined as LSM ≥ 8.6 kPa. Cirrhosis was defined as LSM ≥ 13.1 kPa. Abbreviations: CMRF, cardiometabolc risk factor; FAST, FibroScan-aspartate aminotransferase; LSM, liver stiffness measurement; MASH, metabolic dysfunction–associated steatohepatitis; MASLD, metabolic dysfunction–associated steatotic liver; MetALD; metabolic dysfunction–associated and alcohol-associated liver disease; NH, non-Hispanic; NHANES, National Health and Nutrition Examination Survey.

Among those with MASLD (Figure 3A), Hispanic adults had the highest age-adjusted prevalence of high-risk MASH (18.0%) and advanced fibrosis (21.1%), whereas NH White adults had the highest age-adjusted prevalence of cirrhosis (6.1%). Notably, among Hispanic subgroups, these differences were remarkably higher in other Hispanic adults (Supplemental Table S3, http://links.lww.com/HC9/A666). Among those with MetALD (Figure 3B), NH White adults had the highest age-adjusted prevalence of high-risk MASH (19.3%), advanced fibrosis (19.5%), and cirrhosis (8.1%).

### Burden of social determinants of health

Adults with ALD-predominant MetALD [mean (SD), 2.9 (1.8)] and ALD [mean (SD), 3.3 (1.5)] had an increased number of unfavorable SDOH variables than those with MASLD [mean (SD), 2.1 (1.7)] (Supplemental Figure S1, http://links.lww.com/HC9/A666). Specifically, adults with MASLD-predominant MetALD had a higher prevalence of single living (not married nor living with a partner, 36.6% vs. 26.9%) than those with MASLD (Table 2). Compared to those with MASLD, adults with ALD-predominant MetALD had a higher prevalence of food insecurity (42.1% vs. 27.7%), limited health care access (none or emergency room, 33.0% vs. 16.1%), and single living (not married nor living with a partner, 39.8% vs. 26.9%). Moreover, adults with ALD had higher prevalence of low household income (poverty-income ratio < 130%, 28.1% vs. 18.0%), food insecurity (45.5% vs. 27.7%), limited health care access (none or emergency room, 33.8% vs. 16.1%), no private insurance (57.3% vs. 39.9%), single living (not married nor living with a partner, 44.3% vs. 26.9%), and foreign-born status (29.6% vs. 18.4%) than those with MASLD.

**TABLE 2:** Weighted prevalence of social determinants of health of US adults 20 years or older by SLD subtypes, NHANES 2017–2018

		Weighted, % (95% CI)			
			MetALD^a^		
SDOH domain	Variable	MASLD^b^	MASLD-Pred^c^	ALD-Pred^d^	ALD^e^
Economic stability	Employment status	—	—	—	—
	Unemployed	16.5 (13.1–20.5)	15.0 (83.4–25.4)	14.5 (7.1–27.3)	26.1 (16.0–39.7)
	Household income	—	—	—	—
	PIR < 130%	18.0 (15.7–20.7)	17.0 (11.9–23.8)	28.1 (17.5–41.9)	28.1 (19.0–39.4)^g^
	Food security	—	—	—	—
	Marginal, low, or very low	27.7 (23.9–31.7)	28.4 (21.2–37.0)	42.1 (31.4–53.5)^g^	45.5 (32.7–58.9)^g^
Education access and quality	Education level	—	—	—	—
	High school graduate or less	39.6 (34.5–45.0)	44.3 (36.9–51.9)	49.2 (33.7–64.8)	47.6 (31.3–64.4)
Health care access and quality	Health care access	—	—	—	—
	None or ER	16.1 (12.9–20.0)	17.5 (11.1–26.6)	33.0 (21.6–46.8)^g^	33.8 (20.1–51.0)^g^
	Health insurance type	—	—	—	—
	Public or none	39.9 (34.5–45.6)	35.1 (28.0–43.0)	48.2 (31.8–65.1)	57.3 (45.9–67.9)^g^
Neighborhood and built environment	Home ownership	—	—	—	—
	Rent a home or other arrangement	31.2 (25.2–37.8)	33.1 (24.4–43.0)	43.9 (30.2–58.6)	54.5 (36.5–71.4)^g^
Social and community context	Marital status	—	—	—	—
	Not married/living with a partner	26.9 (23.3–30.8)	36.6 (28.2–46.0)^g^	39.8 (32.2–48.0)^g^	44.3 (34.1–55.0)^g^
N/A	Nativity status	—	—	—	—
	Foreign-born	18.4 (15.7–21.4)	17.7 (11.1–27.0)	23.5 (13.8–37.0)	29.6 (19.3–42.4)^g^
	SDOH score,^f^ mean (SD)	2.1±1.7	2.3±1.5	2.9±1.8^g^	3.3±1.5^g^

a MetALD was defined as (1) hepatic steatosis; (2) ≥ 1 CMRF; and (3) ≥ 2 drinks/day for women and ≥ 3 drinks/day for men.

b MASLD was defined as (1) hepatic steatosis; (2) ≥ 1 CMRF; and (3) < 2 drinks/day for women and < 3 drinks/day for men.

c MASLD-predominant MetALD was defined as MetALD with 2–3 drinks/day for women and 3–4 drinks/day for men.

d ALD-predominant MetALD was defined as MetALD with > 3 drinks/day for women and > 4 drinks/day for men.

e ALD was defined as > 3 drinks/day for women and > 4 drinks/day with hepatic steatosis in the absence of CMRF and/or AST or ALT > 35 IU/L for men and > 25 IU/L for women in the absence of HBV or HCV infection.

f Constructed by assigning a value of 0 for each favorable and 1 for each unfavorable level; a higher number indicates the presence of more unfavorable SDOH. The following variables were included: employment status, household income, food security, education level, health care access, health insurance, home ownership, and marital status.

g *p* < 0.05, MASLD as the reference group.

Abbreviations: ALD, alcohol-associated liver disease; CMRF, cardiometabolic risk factor; ER, emergency room; MASLD, metabolic dysfunction–associated steatotic liver; MetALD, metabolic dysfunction–associated and alcohol-associated liver disease; NHANES, National Health and Nutrition Examination Survey; PIR, poverty-income ratio; SDOH, social determinants of health; SLD, steatotic liver disease.

**FIGURE 4:**
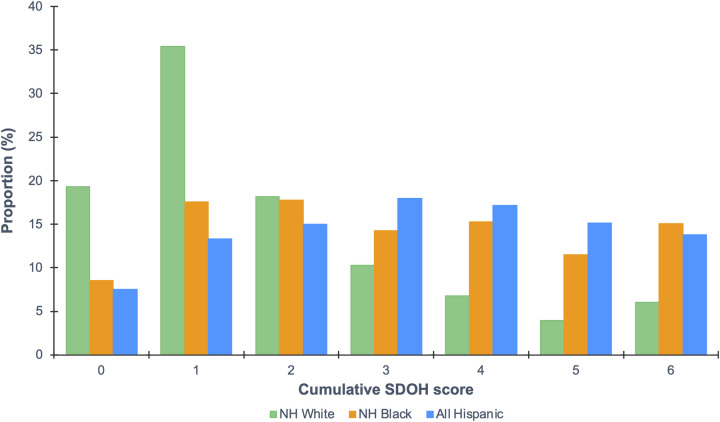
Weighted distribution of cumulative SDOH score among US adults 20 years or older with SLD by race and ethnicity, NHANES 2017–2018. SDOH score was constructed by assigning a value of 0 for each favorable and 1 for each unfavorable level; a higher number indicates the presence of more unfavorable SDOH. The following variables were included: employment status, household income, food security, education level, health care access, health insurance, home ownership, and marital status. Abbreviations: NH, non-Hispanic; NHANES, National Health and Nutrition Examination Survey; SDOH, social determinants of health; SLD, steatotic liver disease.

Across racial and ethnic subgroups, both Hispanic [mean (SD), 3.3 (2.0)] and NH Black [mean (SD), 3.1 (2.6)] adults with SLD had an increased number of unfavorable SDOH than NH White [mean SD, 1.9 (1.2)] (Figure 4). Among Hispanic adults with SLD, differences were more striking in food insecurity (52.2% vs. 21.7%), low education attainment (high school graduate or less, 60.8% vs. 37.4%), limited health care access (none or emergency room, 32.3% vs. 14.5%), no private insurance (54.0% vs. 34.6%), and no home ownership (52.8% vs. 26.9%) when compared with NH White adults (Supplemental Table S4, http://links.lww.com/HC9/A666). Among NH Black adults with SLD, differences were more substantial in unemployment (27.7% vs. 14.7%), low household income (poverty-income ratio < 130%, 31.4% vs. 14.4%), and single living (not married nor living with a partner, 51.9% vs. 26.6%) when compared to NH White adults.

### Associations of race and ethnicity with SLD

In stepwise logistic regression analyses, Hispanic adults (OR 1.49, 95% CI, 1.14–1.1.94) have increased odds of SLD compared with NH White adults after adjusting for age and gender (Table 3). These differences were attenuated after adjusting for diabetes, hypertension, and BMI (OR 1.40, 95% CI, 1.06–1.84) and alcohol use (OR 1.36, 95% CI, 1.01–1.82). Differences did not persist after adjusting for cumulative SDOH and foreign-born status (OR 1.22, 95% CI, 0.89–1.68).

**TABLE 3:** Stepwise regression for potential risk factors for the association between race and ethnicity and odds of SLD among US adults 20 years or older, NHANES 2017–2018

	OR (95% CI)			
SLD^a^	Model 1^b^	Model 2^c^	Model 3^d^	Model 4^e^
Demographics				
Race and ethnicity				
NH White	[Reference]	[Reference]	[Reference]	[Reference]
NH Black	0.73 (0.58–0.94)^g^	0.42 (0.31–0.56)^g^	0.43 (0.32–0.57)^g^	0.42 (0.31–0.57)^g^
All Hispanic	1.49 (1.14–1.94)^g^	1.40 (1.06–1.84)^g^	1.36 (1.01–1.82)^g^	1.22 (0.89–1.68)
NH Other	0.98 (0.67–1.43)	1.15 (0.78–1.69)	1.17 (0.80–1.70)	1.07 (0.75–1.54)
Age, y	1.02 (1.02–1.02)^g^	1.01 (1.00–1.02)^g^	1.02 (1.01–1.02)^g^	1.02 (1.01–1.02)^g^
Gender				
Men	[Reference]	[Reference]	[Reference]	[Reference]
Women	0.62 (0.52–0.75)^g^	0.56 (0.45–0.70)^g^	0.60 (0.48–0.74)^g^	0.60 (0.48–0.74)^g^
Metabolic risks				
Hypertension	—	1.79 (1.46–2.18)^g^	1.76 (1.44–2.16)^g^	1.76 (1.44–2.16)^g^
Diabetes	—	2.79 (1.89–4.12)^g^	2.86 (1.97–4.15)^g^	2.83 (1.96–4.09)^g^
BMI, kg/m^2^	—	1.20 (1.17–1.22)^g^	1.20 (1.17–1.22)^g^	1.20 (1.17–1.22)^g^
Alcohol use risk				
No. of drinks/day	—	—	1.09 (1.01–1.18)^g^	1.09 (1.01–1.18)^g^
Social risks				
SDOH score^f^	—	—	—	1.01 (0.95–1.07)
Nativity status				
US-born	—	—	—	[Reference]
Foreign-born	—	—	—	1.19 (0.84–1.70)

a SLD includes patients with MASLD, MetALD, and ALD.

b Model 1 adjusted for demographic characteristics (age, gender).

c Model 2 adjusted for variables included in model 1 and metabolic risks (hypertension, diabetes, BMI).

d Model 3 adjusted for variables included in model 2 and alcohol consumption.

e Model 4 adjusted for variables included in model 3, SDOH score, and nativity status.

f SDOH score was constructed by assigning a value of 0 for each favorable and 1 for each unfavorable level; a higher number indicates the presence of more unfavorable SDOH. The following variables were included: employment status, household income, food security, education level, health care access, health insurance, home ownership, and marital status.

g *p* < 0.05.

Abbreviations: ALD, alcohol-associated liver disease; MASLD, metabolic dysfunction–associated steatotic liver disease; MetALD, metabolic dysfunction–associated and alcohol-associated liver disease; NH, non-Hispanic; NHANES, National Health and Nutrition Examination Survey; SDOH, social determinants of health; SLD, steatotic liver disease.

In secondary analyses, fully adjusted models revealed increased odds of advanced fibrosis in other Hispanic adults with SLD (OR 2.49, 95% CI, 1.03–6.02) but no differences in odds of high-risk MASH (OR 1.34, 95% CI, 0.65–2.75) (Supplemental Table S5, http://links.lww.com/HC9/A666).

## DISCUSSION

In this nationally representative study of the US population, we defined SLD according to new nomenclature and observed disparities in the prevalence, severity, and burden of SDOH across SLD subtypes and racial and ethnic subgroups. First, we showed that Hispanic adults had the highest prevalence of MASLD, MASLD-predominant MetALD, ALD-predominant MetALD, and ALD. Second, the prevalence of disease severity, namely high-risk MASH, advanced fibrosis, and cirrhosis, was highest among Hispanic adults with MASLD and NH White adults with MetALD. Third, the prevalence of unfavorable SDOH was highest among those with ALD-predominant MetALD and ALD and disproportionately affected Hispanic and NH Black adults. Lastly, the odds of SLD in Hispanic adults were attenuated after adjusting for metabolic risks (hypertension, diabetes, BMI) and alcohol use, and differences did not persist after adjusting for social risks and nativity status.

We found that the prevalence of pure MASLD is 21.6% (44.9 million adults) and MASLD-predominant MetALD is 8.5% (16.9 million adults), consistent with studies showing that the prevalence of NAFLD ranges between 20% and 30%.^3^ We also revealed that the prevalence of ALD-predominant MetALD is 2.6% (5.0 million adults), and ALD is 3.2% (5.9 million adults). These findings are similar to the estimated 4.7% prevalence of ALD in 2015–2016^30,34^ and expected given that (1) MetALD encompasses individuals with both metabolic-associated and alcohol-associated risk factors and (2) the ongoing worsening alcohol-use epidemic in the United States. Furthermore, we demonstrated that Hispanic adults, particularly Mexican Americans, are disproportionately affected by SLD. These results are supported by prior research showing that Hispanic individuals in the United States have a disproportionately high prevalence of NAFLD and ALD.^7,8,30^ It is important to note that there is an increasing recognition that the burden of disease varies among Hispanic subpopulations, as demonstrated in 2 prior studies showing that Mexican-American adults have higher rates of NAFLD compared to those from Puerto Rican or Cuban backgrounds.^35,36^ Disaggregated analyses of Hispanic adults in the United States are urged to fully understand the burden of liver disease for health-policy planning and efficient allocation of resources.

Our study showed that the severity of SLD subtypes varies across racial and ethnic subgroups. Specifically, Hispanic adults with MASLD had the highest prevalence of high-risk MASH and advanced fibrosis, while NH White adults with MetALD had the highest prevalence of high-risk MASH, advanced fibrosis, and cirrhosis. Notably, differences among those with MASLD were mainly driven by the group categorized as other Hispanic and not by those of Mexican origin. A systematic review and meta-analysis evaluating racial and ethnic disparities in NAFLD prevalence and severity in the United States demonstrated that the risk of NASH was greatest among Hispanic individuals; however, the proportion with advanced fibrosis did not differ.^7^ This might be explained by the traditional reporting of Hispanic adults as an aggregated group, which could be masking potential meaningful differences, such as liver fibrosis, the most important predictor of prognosis in patients with NAFLD.^37,38^ Identifying the heterogeneity of disease severity among Hispanic subpopulations is particularly relevant as Hispanic adults experience increased mortality from both cirrhosis and HCC.^39^

Our findings provide insights into the influence of SDOH on SLD and their interplay with Hispanic ethnicity. We observed a higher prevalence of unfavorable SDOH, such as food insecurity, limited health care access, and single living, among adults with ALD-predominant MetALD and ALD compared with those with MASLD. Additionally, our analysis revealed that the associations between Hispanic ethnicity and SLD are not only driven by metabolic risk factors and alcohol use but also mediated by social factors. These findings might be explained by the disproportionate burden of unfavorable SDOH among Hispanic adults compared with NH White adults. Our results are supported by work showing that lower socioeconomic status, including lower education attainment and household income, are risk factors for harmful drinking.^40^ Another study revealed that the incidence rate of ALD increased with the decrease of education level and employment rank among those aged 30–69 years and was higher among those unemployed or receiving disability.^41^ Moreover, a recent study including patients with NAFLD from a tertiary health care system in New York showed that foreign-born status, public health insurance, and lack of transportation were associated with NASH.^13^ Altogether, our study suggests that if we effectively manage medical comorbidities and address SDOH, especially among the most vulnerable populations, we have the potential to mitigate disparities among patients with SLD.

Our study has limitations. First, NHANES is a cross-sectional survey study, and cause-effect inferences cannot be made. Second, despite the oversampling of Hispanic participants, NHANES 2017–2018 categorized this population into 2 groups, Mexican American and other Hispanic adults, limiting subgroup-specific analyses among Hispanic subpopulations. Third, there is potential information bias of the disease severity measures of interest, namely high-risk MASH, advanced fibrosis, and cirrhosis, given no universal cutoff for controlled attenuation parameters and LSM values. Specifically, the use of an LSM threshold of 13.1 kPa to determine the prevalence of cirrhosis may result in an overestimation of the actual occurrence of this liver disease stage. However, we followed methods validated in previous studies. Fourth, alcohol consumption was based on self-report, which could be subject to misclassification bias and underdiagnosis. Nonetheless, this would have biased our results towards the null. Fifth, the timing of data collection does not account for the recent rise in alcohol consumption concurrent with the COVID-19 pandemic.^42,43^ Lastly, we do not have further information on the other Hispanic ethnic groups and community-level SDOH factors, for example, structural racism, were unable to be measured.

### Implications

The new multi-society nomenclature is an opportunity to generate knowledge and promote interdisciplinary interventions in this common group of individuals with metabolic-associated and alcohol-associated risk factors. The United States has been experiencing not only an alcohol-use epidemic, evidenced by the recent increase in alcohol-associated deaths predating and significantly exacerbated by the COVID-19 pandemic,^42^ but also a dramatic increase in the prevalence of severe obesity among both children and adults.^44^ Our data highlight the disproportionate burden of MASLD and MetALD among Hispanic adults. Tackling SLD will require multi-level interventions, such as strategic resource allocation, to screen for and treat alcohol use disorder,^39^ development of culturally tailored education programs aiming to increase food quality,^45^ and implementation of community-level policies such as minimum U pricing which have been shown the greatest impact in health outcomes among the most vulnerable populations.^46,47^

In this nationally representative cross-sectional study, we demonstrated substantial disparities in the prevalence and disease severity of SLD subtypes in the United States, particularly among Hispanic adults. Moreover, the burden of unfavorable SDOH disproportionately affected those with ALD-predominant MetALD and ALD. Notably, the associations between Hispanic ethnicity and SLD were not only attenuated by metabolic risks and alcohol use but also by an increasing number of adverse social factors and nativity status. Our findings emphasize the important role of unfavorable SDOH in the burden of SLD among US adults. Further studies are needed to evaluate population-based approaches targeting SDOH, such as policy on food quality and alcohol availability, as these factors may mitigate race and ethnic differences.

## Supplementary Material

SUPPLEMENTARY MATERIAL
