# Supplementary material for: Racial and ethnic differences and the role of unfavorable social determinants of health across steatotic liver disease subtypes in the United States
Source: Hepatol Commun. 2023 Dec 1;7(12):e0324. doi: 10.1097/HC9.0000000000000324 (PMC10697602; doi:10.1097/HC9.0000000000000324)
Supplement: SUPPLEMENTARY MATERIAL [file hc9-7-e0324-s001.docx]

**SUPPLEMENTAL ONLINE CONTENT**

**Supplemental Figure 1.** Weighted Distribution of Cumulative SDOH Variables Among U.S. Adults 20 Years or Older by Steatotic Liver Disease Subtypes, NHANES 2017-2018.

**Supplemental Table 1.** Weighted Characteristics of U.S. Adults 20 Years or Older by Race and Ethnicity, NHANES 2017-2018.

**Supplemental Table 2.** Population Estimate, Weighted Crude and Age-Adjusted Prevalence of Hepatic Steatosis and Steatotic Liver Disease Subtypes Among U.S. Adults 20 Years or Older by Race and Ethnicity, NHANES 2017-2018.

**Supplemental Table 3.** Weighted Age-Adjusted Prevalence of High-Risk MASH, Advanced Fibrosis, and Cirrhosis Among U.S. Adults 20 Years or Older with MASLD and MetALD by Race and Ethnicity, NHANES 2017-2018

**Supplemental Table 4.** Weighted Distribution of Social Determinants of Health of U.S. Adults 20 Years or Older by Race and Ethnicity, NHANES 2017-2018

**Supplemental Table 5.** Weighted Multivariable Logistic Regression for High-Risk MASH and Advanced Fibrosis Among U.S. Adults 20 Years or Older with Steatotic Liver Disease, NHANES 2017-2018.

**Supplemental Figure 1.** Weighted Distribution of Cumulative SDOH Score Among U.S. Adults 20 Years or Older by Steatotic Liver Disease Subtypes, NHANES 2017-2018.

**
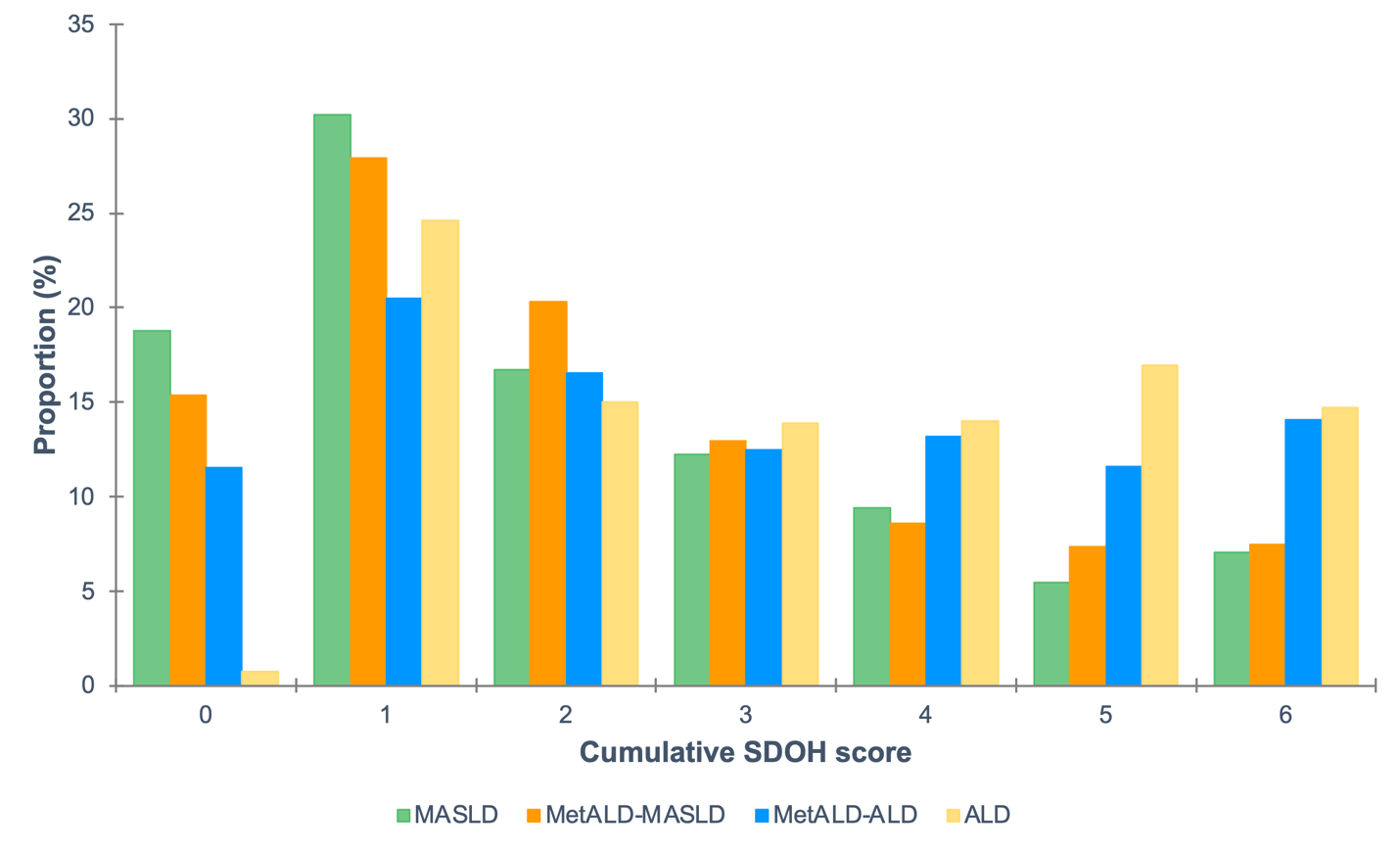
**

Abbreviations: ALD, alcohol-associated liver disease; CAP, controlled attenuation parameter; MASLD, metabolic dysfunction-associated steatotic liver; MetALD; metabolic dysfunction- and alcohol-associated liver disease; NHANES, National Health and Nutrition Examination Survey; U.S., United States.

Constructed by assigning a value of 0 for each favorable and 1 for each unfavorable level; a higher number indicates the presence of more unfavorable SDOH. The following variables were included: employment status, household income, food security, education level, healthcare access, health insurance, home ownership, and marital status. MASLD was defined as (1) hepatic steatosis; (2) ≥ 1 metabolic risk factor; and (3) < 2 drinks/day for women and < 3 drinks/day for men. MetALD was defined as (1) hepatic steatosis; (2) ≥ 1 metabolic risk factor; and (3) ≥ 2 drinks/day for women and ≥ 3 drinks/day for men. MASLD-predominant MetALD was defined as MetALD with 2-3 drinks/day for women and 3-4 drinks/day for men. ALD-predominant MetALD was defined as MetALD with > 3 drinks/day for women and > 4 drinks/day for men. ALD was defined as > 3 drinks/day for women and > 4 drinks/day with hepatic steatosis in the absence of CMRF and/or AST or ALT > 35 IU/L for men and > 25 IU/L for women in the absence of HBV or HCV infection.

**Supplemental Table 1**. Weighted Characteristics of U.S. Adults 20 Years or Older with Steatotic Liver Disease by Race and Ethnicity, NHANES 2017-2018

|  | **Weighted, % (95% CI)** | | | | |
| --- | --- | --- | --- | --- | --- |
| **Steatotic Liver Disease^a^** | **NH White** | **NH Black** | **All Hispanic** | **Mexican American** | **Other Hispanic** |
| **Demographics** |  |  |  |  |  |
| Population Estimate | 46,248,671 | 6,179,088 | 13,356,001 | 9,007,470 | 4,348,531 |
| Age, mean (SD), year | 54 ± 11 | 50 ± 18 | 44 ± 17 | 42 ± 16 | 48 ± 17 |
| Age, group, year |  |  |  |  |  |
| 20-39 | 21.8 (17.0-27.5) | 24.6 (17.0-34.4) | 42.5 (35.0-50.3) | 46.9 (38.3-55.6) | 33.4 (24.9-43.1) |
| 40-59 | 38.0 (30.2-46.4) | 51.4 (41.2-61.5) | 38.8 (32.7-45.3) | 39.2 (32.6-46.1) | 38.0 (27.9-49.2) |
| ≥60 | 40.2 (33.0-47.9) | 24.0 (19.3-29.4) | 18.7 (13.5-25.4) | 14.0 (9.4-20.5) | 28.6 (21.0-37.7) |
| Gender, women | 43.8 (39.1-48.6) | 51.7 (44.0-59.4) | 40.1 (33.8-46.9) | 37.5 (31.1-44.4) | 45.6 (35.9-55.6) |
| **Risk factors** |  |  |  |  |  |
| Cigarette smoking | 15.9 (13.0-19.5) | 24.9 (20.5-29.9) | 15.0 (9.2-23.5) | 18.6 (10.7-30.3) | 7.6 (4.0-14.0) |
| No. drinks/day, mean (SD) | 1.9 ± 1.4 | 1.7 ± 2.1 | 3.1 ± 3.3 | 3.4 ± 3.4 | 2.4 ± 2.9 |
| Dyslipidemia | 80.8 (73.4-86.5) | 71.9 (65.9-77.3) | 81.8 (76.4-86.3) | 81.3 (73.8-87.1) | 82.9 (65.6-92.5) |
| Hypertension | 71.4 (64.6-77.3) | 77.2 (69.9-83.1) | 54.6 (48.9-60.2) | 50.8 (44.0-57.4) | 62.7 (55.5-69.4) |
| Diabetes | 25.5 (20.7-30.9) | 30.3 (26.3-34.6) | 22.6 (18.7-27.0) | 20.3 (16.2-25.1) | 27.3 (17.8-39.5) |
| BMI, mean (SD), kg/m^2^ | 34.2 ± 4.7 | 36.3 ± 10.2 | 33.5 ± 6.2 | 34.0 ± 6.3 | 32.5 ± 5.7 |
| Waist circumference, mean (SD), cm | 114 ± 10 | 114 ± 20 | 109 ± 13 | 110 ± 13 | 108 ± 14 |
| HOMA-IR | 5.5 ± 5.2 | 6.4 ± 8.9 | 8.1 ± 19.0 | 8.6 ± 20.9 | 6.9 ± 11.5 |
| AST, mean (SD), IU/L | 24 ± 9 | 25 ± 23 | 25 ± 17 | 25 ± 16 | 27 ± 20 |
| ALT, mean (SD), IU/L | 28 ± 13 | 26 ± 26 | 33 ± 27 | 32 ± 25 | 34 ± 41 |
| Total energy, mean (SD), kcal/day | 2,193 ± 559 | 1,929 ± 930 | 2,152 ± 907 | 2,128 ± 885 | 2,195 ± 934 |
| Total sugar, mean (SD), gm | 105 ± 44 | 98 ± 65 | 111 ± 87 | 106 ± 65 | 119 ± 112 |
| CAP, mean (SD), dB/m | 330 ± 26 | 324 ± 48 | 326 ± 39 | 324 ± 38 | 330 ± 40 |
| LSM, mean (SD), kPa | 7.1 ± 4.7 | 6.4 ± 4.1 | 6.6 ± 5.0 | 6.4 ± 3.8 | 7.1 ± 6.9 |
| FAST score, mean (SD) | 0.19 ± 0.12 | 0.18 ± 0.23 | 0.20 ± 0.21 | 0.19 ± 0.20 | 0.22 ± 0.21 |
| FIB-4 score > 2.67 | 2.5 (1.7-3.7) | 3.2 (1.4-6.9) | 2.2 (1.2-4.1) | 1.4 (0.6-3.5) | 3.9 (1.8-8.3) |
| NFS score > 0.676 | 14.0 (11.8-16.6) | 18.5 (15.6-22.0) | 8.0 (5.3-11.9) | 6.6 (4.4-10.0) | 10.7 (5.6-19.7) |

Abbreviations: ALD, alcohol-associated liver disease; ALT, alanine aminotransferase; AST, aspartate aminotransferase; BMI, body mass index; CAP, controlled attenuation parameter; FAST, FibroScan-AST; FIB-4; fibrosis-4; HOMA-IR, Homeostatic Model Assessment for Insulin Resistance; LSM, liver stiffness measurement; MASLD, metabolic dysfunction-associated steatotic liver; MetALD, metabolic dysfunction- and alcohol-associated liver disease; NFS, NAFLD fibrosis score, NH, non-Hispanic; NHANES, National Health and Nutrition Examination Survey; U.S., United States.

^a^ Steatotic liver disease includes patients with MASLD, MetALD, and ALD.

**Supplemental Table 2.** Population Estimate, Weighted Crude and Age-Adjusted Prevalence of Hepatic Steatosis and Steatotic Liver Disease Subtypes Among U.S. Adults 20 Years or Older by Race and Ethnicity, NHANES 2017-2018

|  | **No. of** | **Population** | **Crude** | **Age-Adjusted** | **Rate** |
| --- | --- | --- | --- | --- | --- |
| **Race and Ethnicity** | **Cases^a^** | **Estimate** | **Prevalence, %** | **Prevalence, %^b^** | **Ratio^c^** |
| **Hepatic steatosis^d^** |  |  |  |  |  |
| All Adults | 1,621 | 72,364,080 | 36.3 | 35.7 |  |
| NH White | 598 | 45,968,708 | 36.6 | 35.1 |  |
| NH Black | 292 | 5,930,546 | 26.9 | 27.5 | 0.78 |
| All Hispanic | 452 | 13,653,126 | 43.5 | 44.4 | 1.26 |
| Mexican American | 306 | 9,263,342 | 52.2 | 52.4 | 1.49 |
| Other Hispanic | 146 | 4,389,783 | 32.2 | 33.6 | 0.96 |
| **MASLD^e^** |  |  |  |  |  |
| All Adults | 1092 | 44,888,618 | 22.5 | 21.6 |  |
| NH White | 418 | 29,750,252 | 23.7 | 21.7 |  |
| NH Black | 207 | 3,990,620 | 18.1 | 18.6 | 0.86 |
| All Hispanic | 246 | 6,382,888 | 20.3 | 22.3 | 1.02 |
| Mexican American | 155 | 3,813,887 | 21.5 | 24.0 | 1.11 |
| Other Hispanic | 91 | 2,569,001 | 18.8 | 20.1 | 0.93 |
| **MetALD^f^** |  |  |  |  |  |
| All Adults | 420 | 21,959,169 | 11.0 | 11.2 |  |
| NH White | 144 | 13,530,081 | 10.8 | 10.9 |  |
| NH Black | 77 | 1,708,648 | 7.8 | 7.9 | 0.72 |
| All Hispanic | 153 | 5,154,117 | 16.4 | 15.9 | 1.46 |
| Mexican American | 111 | 3,917,034 | 22.1 | 20.6 | 1.89 |
| Other Hispanic | 42 | 1,237,084 | 9.1 | 9.3 | 0.85 |
| **MetALD-MASLD^g^** |  |  |  |  |  |
| All Adults | 307 | 16,897,094 | 8.5 | 8.5 |  |
| NH White | 101 | 10,657,502 | 8.5 | 8.6 |  |
| NH Black | 67 | 1,479,103 | 6.7 | 6.8 | 0.79 |
| All Hispanic | 96 | 3,258,267 | 10.4 | 10.3 | 1.20 |
| Mexican American | 65 | 2,330,429 | 13.1 | 12.6 | 1.47 |
| Other Hispanic | 31 | 927,838 | 6.8 | 7.0 | 0.81 |
| **MetALD-ALD^h^** |  |  |  |  |  |
| All Adults | 113 | 5,062,075 | 2.5 | 2.6 |  |
| NH White | 43 | 2,872,580 | 2.3 | 2.3 |  |
| NH Black | 10 | 229,545 | 1.0 | 1.0 | 0.43 |
| All Hispanic | 57 | 1,895,851 | 6.0 | 5.6 | 2.43 |
| Mexican American | 46 | 1,586,605 | 8.9 | 8.0 | 3.48 |
| Other Hispanic | 11 | 309,246 | 2.3 | 2.2 | 0.96 |
| **ALD^i^** |  |  |  |  |  |
| All Adults | 112 | 5,892,916 | 3.0 | 3.2 |  |
| NH White | 36 | 2,968,337 | 2.4 | 2.7 |  |
| NH Black | 14 | 479,820 | 2.2 | 2.1 | 0.78 |
| All Hispanic | 50 | 1,818,995 | 5.8 | 5.4 | 2.00 |
| Mexican American | 36 | 1,276,549 | 7.2 | 6.5 | 2.41 |
| Other Hispanic | 14 | 542,446 | 4.0 | 3.9 | 1.44 |

Abbreviations: ALD, alcohol-associated liver disease; CAP, controlled attenuation parameter; CMRF, cardiometabolic risk factor; MASLD, metabolic dysfunction-associated steatotic liver; MetALD; metabolic dysfunction- and alcohol-associated liver disease; NH, non-Hispanic; NHANES, National Health and Nutrition Examination Survey; U.S., United States.

^a^ Unweighted total number of participants. ^b^ Estimates were standardized to the 2000 U.S. Census population. ^c^ Rate ratios relative to NH White adults. ^d^ Hepatic steatosis was defined as a CAP ≥ 285 dB/m. ^e^ MASLD was defined as (1) hepatic steatosis; (2) ≥ 1 CMRF; and (3) < 2 drinks/day for women and < 3 drinks/day for men. ^f^ MetALD was defined as (1) hepatic steatosis; (2) ≥ 1 CMRF; and (3) ≥ 2 drinks/day for women and ≥ 3 drinks/day for men. ^g^ MASLD-predominant MetALD was defined as MetALD with 2-3 drinks/day for women and 3-4 drinks/day for men. ^h^ ALD-predominant MetALD was defined as MetALD with > 3 drinks/day for women and > 4 drinks/day for men. ALD was defined as > 3 drinks/day for women and > 4 drinks/day with hepatic steatosis in the absence of CMRF and/or AST or ALT > 35 IU/L for men and > 25 IU/L for women in the absence of HBV or HCV infection.

**Supplemental Table 3.** Weighted Age-Adjusted Prevalence of High-Risk MASH, Advanced Fibrosis, and Cirrhosis Among U.S. Adults 20 Years or Older with MASLD and MetALD by Race and Ethnicity, NHANES 2017-2018

|  | **Age-Adjusted Prevalence, %** | | |
| --- | --- | --- | --- |
| **Race and Ethnicity** | **High-Risk MASH^a^** | **Advanced Fibrosis^b^** | **Cirrhosis^c^** |
| **MASLD^d^** |  |  |  |
| All Adults | 14.6 | 15.6 | 5.5 |
| NH White | 14.3 | 14.6 | 6.1 |
| NH Black | 9.8 | 16.5 | 5.6 |
| All Hispanic | 18.0 | 21.1 | 4.8 |
| Mexican American | 16.5 | 16.1 | 3.8 |
| Other Hispanic | 19.9 | 28.4 | 6.4 |
| **MetALD^e^** |  |  |  |
| All Adults | 14.4 | 14.3 | 5.5 |
| NH White | 19.3 | 19.5 | 8.1 |
| NH Black | 13.8 | 5.6 | 1.9 |
| All Hispanic | 7.7 | 9.2 | 2.4 |
| Mexican American | 4.2 | 6.9 | 1.1 |
| Other Hispanic | 18.8 | 13.6 | 3.2 |

Abbreviations: CMRF, cardiometabolc risk factor; FAST, FibroScan-AST; LSM, liver stiffness measurement; MASH, metabolic dysfunction-associated steatohepatitis; MASLD, metabolic dysfunction-associated steatotic liver; MetALD; metabolic dysfunction- and alcohol-associated liver disease; NH, non-Hispanic; NHANES, National Health and Nutrition Examination Survey; U.S., United States.

^a^ High-risk MASH was defined as FAST score ≥ 0.35. ^b^ Advanced fibrosis was defined as LSM ≥ 8.6 kPa. ^c^ Cirrhosis was defined as LSM ≥ 13.1 kPa. ^d^ MASLD was defined as (1) hepatic steatosis; (2) ≥ 1 CMRF; and (3) < 2 drinks/day for women or < 3 drinks/day for men. ^e^ MetALD was defined as (1) hepatic steatosis; (2) ≥ 1 CMRF; and (3) ≥ 2 drinks/day for women or ≥ 3 drinks/day for men.

**Supplemental Table 4.** Weighted Distribution of Social Determinants of Health of U.S. Adults 20 Years or Older with Steatotic Liver Disease by Race and Ethnicity, NHANES 2017-2018

|  |  | **Weighted, % (95% CI)** | |  |  |  |
| --- | --- | --- | --- | --- | --- | --- |
| **SDOH Domain** | **Variable** | **NH White** | **NH Black** | **All Hispanic** | **Mexican American** | **Other Hispanic** |
| Economic Stability | Employment status |  |  |  |  |  |
|  | Unemployed | 14.1 (10.8-19.7) | 27.7 (21.2-35.3) | 18.6 (14.5-23.6) | 18.3 (13.2-24.7) | 19.4 (15.2-24.4) |
|  | Household income |  |  |  |  |  |
|  | PIR < 130% | 14.4 (11.7-17.6) | 31.4 (26.8-36.5) | 30.5 (23.5-38.5) | 32.2 (24.1-41.5) | 26.8 (18.6-37.1) |
|  | Food security |  |  |  |  |  |
|  | Marginal, low, or very low | 21.7 (18.0-26.0) | 39.5 (33.4-46.0) | 52.2 (41.6-62.6) | 51.5 (38.9-63.9) | 53.6 (41.3-65.5) |
| Education Access  and Quality | Education level |  |  |  |  |  |
|  | High school graduate or less | 37.4 (33.9-41.1) | 46.2 (37.8-54.9) | 60.8 (53.1-68.0) | 65.0 (55.2-73.7) | 52.1 (40.9-63.1) |
| Healthcare Access  and Quality | Healthcare access |  |  |  |  |  |
|  | None or ER | 14.5 (11.1-18.8) | 22.9 (13.9-35.4) | 32.3 (24.1-41.8) | 35.8 (26.1-46.9) | 25.0 (16.7-35.7) |
|  | Health insurance type |  |  |  |  |  |
|  | Public or none | 34.6 (29.6-39.9) | 53.1 (43.8-62.2) | 54.0 (47.1-60.8) | 53.7 (45.4-61.8) | 54.7 (40.4-68.3) |
| Neighborhood and  Built Environment | Home ownership |  |  |  |  |  |
|  | Rent or other arrangement | 26.9 (20.2-34.9) | 46.6 (38.4-54.9) | 52.8 (42.9-62.4) | 54.4 (43.0-65.3) | 49.5 (38.3-60.8) |
| Social and  Community Context | Marital status |  |  |  |  |  |
|  | Not married/living with partner | 26.6 (21.9-31.9) | 51.9 (42.8-60.8) | 37.9 (32.8-43.2) | 39.3 (32.3-46.7) | 34.9 (25.2-46.1) |
| N/A | Nativity status |  |  |  |  |  |
|  | Foreign born | 3.7 (2.2-6.3) | 7.0 (5.0-9.8) | 61.9 (54.0-69.2) | 59.6 (49.3-69.2) | 66.7 (57.0-75.1) |
|  | SDOH score,^f^ mean (SD) | 1.9 ± 1.2 | 3.1 ± 2.6 | 3.3 ± 2.0 | 3.4 ± 2.0 | 3.0 ± 2.1 |

Abbreviations: ER, emergency room; NHANES, National Health and Nutrition Examination Survey; PIR, poverty-income ratio; SDOH, social determinants of health; U.S., United States.

Constructed by assigning a value of 0 for each favorable and 1 for each unfavorable level; a higher number indicates the presence of more unfavorable SDOH. The following variables were included: employment status, household income, food security, education level, healthcare access, health insurance, home ownership, and marital status.

**Supplemental Table 5.** Weighted Multivariable Logistic Regression for High-Risk MASH and Advanced Fibrosis Among U.S. Adults 20 Years or Older with Steatotic Liver Disease, NHANES 2017-2018.

|  | **OR (95% CI)** | |
| --- | --- | --- |
| **Steatotic Liver Disease^a^** | **High-Risk MASH^b^** | **Advanced Fibrosis^c^** |
| **Demographics** |  |  |
| Race and Ethnicity |  |  |
| NH White | [Reference] | [Reference] |
| NH Black | 0.73 (0.43-1.26) | 0.66 (0.39-1.12) |
| Mexican American | 0.68 (0.31-1.50) | 1.17 (0.47-2.91) |
| Other Hispanic | 1.34 (0.65-2.75) | 2.49 (1.03-6.02)* |
| NH Other | 1.17 (0.68-2.02) | 0.97 (0.53-1.75) |
| Age, year | 0.98 (0971-1.00) | 1.01 (0.99-1.03) |
| Gender |  |  |
| Men | [Reference] | [Reference] |
| Women | 0.47 (0.31-0.72)* | 0.44 (0.21-0.90)* |
| **Metabolic risks** |  |  |
| Hypertension | 1.78 (0.94-3.35) | 1.10 (0.59-2.03) |
| Diabetes | 1.81 (1.19-2.77)* | 2.52 (1.48-4.29)* |
| BMI, kg/m^2^ | 1.06 (1.03-1.10)* | 1.15 (1.12-1.18)* |
| **Alcohol use risk** |  |  |
| No. of drinks/day | 1.10 (1.00-1.21) | 0.98 (0.87-1.10) |
| **Social risks** |  |  |
| SDOH score^d^ | 1.10 (0.97-1.25) | 1.00 (0.93-1.07) |
| Nativity status |  |  |
| U.S. born | [Reference] | [Reference] |
| Foreign born | 1.13 (0.56-2.27) | 0.98 (0.59-1.65) |

Abbreviations: BMI, body mass index; MASH, metabolic dysfunction-associated steatohepatitis; NH, non-Hispanic; NHANES, National Health and Nutrition Examination Survey; SDOH, social determinants of health; U.S., United States.

^a^ Steatotic liver disease includes patients with MASLD, MetALD, and ALD. ^b^ High-risk MASH was defined as FAST score ≥ 0.35. ^c^ Advanced fibrosis was defined as LSM ≥ 8.6 kPa. ^d^ SDOH score was constructed by assigning a value of 0 for each favorable and 1 for each unfavorable level; a higher number indicates the presence of more unfavorable SDOH. The following variables were included: employment status, household income, food security, education level, healthcare access, health insurance, home ownership, and marital status. * *P* <.05
